# A novel dual-target Septin9 methylation assay for improved detection of early-stage colorectal cancer and high-grade intraepithelial neoplasia

**DOI:** 10.1186/s12885-024-12645-4

**Published:** 2024-07-30

**Authors:** Youming Wu, Yongqing Tong, Haitao Zhang, Yun Li, Xu Zhu, Ming Li, Lili Qiu, Wenlan Liu, Siqing Mei, Yu Mao, Yanhua Cao, Caiyan Su, Wentao Yu, Junli Wang, Taizhong Wang, Zhongyuan Zhu, De-Hua Yu

**Affiliations:** 1 Zhongshan Xiaolan People’s Hospital, Zhongshan, China; 2 Clinical Laboratory, Renmin Hospital of Wuhan University, Wuhan, China; 3 Department of Gastrointestinal Surgery, The Second People’s Hospital of Shenzhen, Shenzhen, China; 4 Clinical Laboratory, The Second Affiliated Hospital of Hainan Medical University, Haikou, China; 5 USK Bioscience, Co., Ltd, 5th Floor, Building A, Guanlan High-tech Park, Shenzhen, China; 6 Youjiang Medical University For Nationalities, Baise, Guangxi China

**Keywords:** Colorectal cancer (CRC), Methylation, PCR, Septin9, CpG-rich subregions

## Abstract

**Background:**

Colorectal cancer (CRC) ranks as the third most common malignancies in the world, and periodic examination of the patient is advantageous in reducing the mortality of CRC. The first blood-based Septin9 gene methylation assay which recognized by the US FDA for CRC examination was Epi proColon. However, this assay was not broadly applied in the current clinical guideline because of its relatively lower sensitivity in the detection of early-stage CRC.

**Methods:**

This study aimed at developing a new multiplex Septin9 methylation assay (ColonUSK) which simultaneously evaluates two CpG-rich subregions in the promoter of the Septin9 gene and an internal control in a single reaction. ColonUSK proved increased sensitivity, with a detection limit as low as 12pg of the positive DNA compared with the Septin9 assay targeting one CpG-rich subregion. 1366 subjects were prospectively recruited from four comprehensive hospitals in China in an opportunistic screening study for assessing its value in CRC detection. Blind testing was developed to evaluate ColonUSK in comparison with clinical examination using clinical gold standard such as colonoscopy.

**Results:**

The assay demonstrates clinical sensitivity for diagnosing colorectal cancer (CRC) and advanced adenoma at rates of 77.34% and 25.26%, respectively. Furthermore, ColonUSK exhibits a high degree of specificity for non-CRC cases (95.95%) clinically. Significantly, the detection rate of cases in high-grade intraepithelial neoplasia increased to 54.29%. The value for the assay in the Kappa test was 0.76, showing a high degree of consistency between ColonUSK and clinical gold standard.

**Conclusions:**

ColonUSK indicated moderate diagnostic value and could become a non-invasive detection way for CRC. The implementation of the ColonUSK assay has the capacity to markedly enhance CRC screening practices.

**Supplementary Information:**

The online version contains supplementary material available at 10.1186/s12885-024-12645-4.

## Background

Colorectal cancer (CRC) remians the third most common cancer in the world, acounting for estimated 10% of all tumour cases. There were an approximately 1.9 million new cases of CRC and the quantity of mortality reached to 935,000 globally in 2020 [1–3]. The incidence and mortality rates of CRC vary across regions and populations, with factors such as age, diet, lifestyle, and genetic predisposition influencing the risk of development.

In China, CRC poses a significant health challenge. According to the National Cancer Center of China, CRC remians second and fifth in terms of the highest incidence and death rates among all malignancies [4]. In 2020 alone, new cases and deaths of CRC were approximately 555,477 and 367,984 respectively. Notably, China has witnessed a consistent rise in the incidence of CRC in the past three decades [5]. From 1990 to 2016, the age-standardized incidence rate (ASIR) of CRC mounted up from 14.25 per 100,000 to 25.27 per 100,000, with an annual increase of ASIR by 2.76% in males and 1.70% in females [5]. This trend may be attributed to changes in lifestyle, including the adoption of a more Westernized diet, reduced physical activity, increased smoking rates, and a growing aging population [6, 7].

The development of CRC is a slow course, typically taking several years to progress from dysplasia and adenomas to carcinoma. Consequently, regular screening has the potential to decrease CRC mortality by detecting precancerous polyps or early-stage CRC when these lesions can be removed or are more treatable, respectively. This assumption is strongly supported by a wealth of evidence. First, in the United States, the prevalence of colonoscopy screening for CRC among adults aged 50 and the elderly tripled from 20% in 2000 to 61% in 2018, driven by expanded health insurance coverage initiated in 2001 [8]. This increased capacity for CRC prevention correlated with a significant decline in incidence, about 3–5% annually, among adults aged 50 and older in the late 2000s [9]. Additionally, numerous clinical studies have shown that individuals who undergo screening colonoscopy have a 29–68% lower estimated cancer mortality compared to those who do not [10]. Consistently, it has been observed that either one or two rounds of flexible sigmoidoscopy screening or multiple rounds of biennial screening with fecal occult blood testing (FOBT) had lower CRC-specific deaths compared to no screening controls [11]. Furthermore, using the MISCAN-Colon model, Edwards et al. indicated that CRC incidence in USA declined from 1975 to 2000, with 50% attributed to changes in risk factors and another 50% attributed to screening [12]. The reasons for the decrease in mortality include 35% attributed to changes in risk factors, 53% attributed to screening, and the remaining 12% attributed to treatment [10].

Based on extensive research and evidence supporting the advantage of CRC screening in decreasing morbidity, mortality, and improving outcomes, several organizations, such as the U.S. Preventive Services Task Force (USPSTF) and the American Cancer Society, publish issued guidelines recommending regular CRC screening with various methodologies for average-risk individuals starting at age 45 or 50 [13]. There are some regular screening tests for CRC include colonoscopy such as fecal occult blood testing (FOBT), fecal immunochemical testing (FIT), multi target stool DNA (mtsDNA) testing, and the blood-based Septin9 methylation assay.

The SEPT9 gene, also known as Septin9, plays a significant role in various cellular courses, including cytokinesis, cytoplasmic division, cell polarization, vesicle transport, and membrane reconstruction [14]. This gene encodes the septin-9 protein, which functions as a tumor suppressor. At the molecular level, the transcriptional activity of the SEPT9 gene is regulated by the methylation status of its CpG islands. In its unmethylated state, these CpG islands facilitate active transcription, enabling the SEPT9 gene to exert its tumor-suppressive functions. In contrast, methylation of these islands leads to epigenetic inactivation of the SEPT9 gene, a pivotal event in the pathogenesis of cellular abnormalities [15]. Methylation of the SEPT9 gene is a critical epigenetic alteration that drives cellular progression towards malignancy, particularly in the colon mucosa, emphasizing the importance of epigenetic regulation in oncogenesis [16].

Epi proColon, a blood-based Septin9 gene methylation assay, was approved by the US FDA in 2016 as a CRC screening test for the average-risk population aged over 50 who are unwilling or unable to undergo colonoscopy or other recommended CRC screenings [17]. The effectiveness of methylated Septin9 for CRC detection has been reviewed in the newly published recommendation statement by USPSTF. The blood-based Septin9 gene methylation assay is more economical and has higher compliance compared to stool DNA tests. It has been demonstrated to be an convenient, fast, reliable, and accurate molecular test. However, the broad application of the assay has been hindered by its relatively low sensitivity for CRC. In the present study, we developed a new Septin9 gene methylation assay by increasing detection sites and demonstrated improved capacity.

## Methods

### Participants and plasma specimen collection

Study subjects were prospectively recruited from four comprehensive hospitals in China, including Renmin Hospital of Wuhan University, the Second People’s Hospital of Shenzhen, Zhongshan Xiaolan People’s Hospital, and the Second Affiliated Hospital of Hainan Medical University. A total of 1,366 subjects diagnosed with CRC, adenomas, polyps, normal controls, and other types of digestive diseases and tumors were enrolled in the study. All subjects were identified and confirmed through endoscopic and histopathologic examinations. We excluded patients who had previously treated CRC or other malignancies, and those without complete information. Tumor staging was classified based on the 2021 Guidelines of the Chinese Society of Clinical Oncology (CSCO) for CRC. Histologic differentiation was graded according to the 5th edition of the World Health Organization (WHO) classification of tumors [18].

The plasma specimens were collected from patients with colorectal cancer and other diseases before any surgical treatment, chemotherapy, or radiation therapy, between March 2018 and November 2022. Clinical and pathologic data for patients with CRC and other diseases were collected from the electronic medical record system and through histopathologic review. Participants and samples lacked essential details, specifically concerning their past colorectal cancer surgeries, were not included in the analysis. Participants has other history of neoplasms, or any chemo or radio therapy, and pregnant women were not included. A total of 30 FFPE samples used for validation were provided by Zhongshan Xiaolan People’s Hospital.These samples consisting of 15 CRC tumor tissue samples and 15 paired adjacent normal tissue samples were collected post-Endoscopic Submucosal Dissection (ESD) surgery and had obtained prior informed consent. All participating patients in the research offered written consent. 15 cases were diagnosed as CRC. This research obtained support from the Institutional Review Boards of Renmin Hospital of Wuhan University, the Second People’s Hospital of Shenzhen, and the Second Affiliated Hospital of Hainan Medical University. Written consent was received from all patients.

### Specimen preparation

Venous blood samples were collected in BD Vacutainer^®^ K2EDTA tubes (Becton, Dickinson and Company, Cat#8) and stored at 2–8 °C. They were disposed within 24 h after collection. Before centrifugation, the whole-blood samples stored at 2–8 °C were required to be held at 15 to 30 °C for approximately 30 min. All blood samples were then centrifuged at room temperature at 1500 g for 10 min and re-centrifuged at 1500 g for 10 min in 15 ml tubes. The plasma specimens were carefully transferred to new tubes, avoiding contact with the hemocyte layer, and stored at − 70 °C prior to analysis. Approximately 3 ml plasma was obtained for experiment. DNA was obtained using the Udx ctDNA kit (No. 20180568, USKbio Co.,Ltd China). The patient specimens, along with one positive and one negative control included in the Septin9 Gene Methylation Detection Kit (PCR Fluorescence Probe Method, No. 2023400151 USKbio Co.,Ltd China), were processed with the Udx ctDNA kit.

### DNA extraction and Bisulfite Conversion

DNA from FFPE samples was extracted using FFPE DNA Extraction Kit (Spin Column) (No. 20180134,USKbio Co.,Ltd China) according to the instruction. Briefly, paraffin of the sample is dissolved in xylene and discarded. Then the compound is lysed under denaturing conditions with proteinase K digestion at 56 °C for 1 h. The compound is incubated at 90 °C for reversing DNA and protein crosslinking. DNA binds to the membrane of Spin Column and contaminants are removed. The washing buffer is used to deal with Other contaminants. DNA is collected in the elution buffer and stored at − 20 °C.

DNA from the plasma specimens was extracted using Udx ctDNA kit (No. 20180568, USKbio Co.,Ltd China). In the instruction, 3 mL of plasma was digested with 3.75 mL of lysis buffer at 60 °C for 20 min and DNA was extracted using magnetic particles, which captures the nucleic acids, while unbound components were washing away.

Following the manufacturer’s protocol, bisulfite modification was carried out using DNA Bisulfite Conversion Kit (No. 20180516, USKbio Co., Ltd China). 30 µl of DNA protection buffer and 70 µl of bisulfite mix in the DNA Bisulfite Conversion Kit were combined with 50 µl of the DNA sample. The mixture was then placed in a thermal cycler and subjected to the following amplification conditions: 95 °C for 3 min; 3 cycles of 95 °C for 1 min, 95 °C for 1 min and 80 °C for 10 min. The cycle concluded with a hold at 25 °C. Subsequently, the bisulfite-converted DNA was mixed with BS-binding buffer, and desulfurization, washing, and elution were carried out using magnetic particles with 17 µl of water.

### Real-time PCR for detecting methylated SEPT9

Bisulfte-converted DNA template are amplifed by using Septin9 Gene Methylation Detection Kit (PCR Fluorescence Probe Method, No. 2023400151 USKbio Co.,Ltd China). In the instruction, a single 50 µL PCR containing 24.5 µL of reaction buffer, 0.5 µL of PCR polymerase, and 25 µL of bis-DNA are performed. The methylated and unmethylated DNAs can be differentiated in this process. Fluorescence FAM for Septin9 and fluorescence JOE for ACTB were used to bind different probes targeting specific gene methylation. The thermal cycling program: 94 °C for 5 min; 45 cycles of 62 °C for 5 s, 58 °C for 35 s and 93 °C for 30 s; 40 °C for 5 s using real-time PCR (RT-PCR) systems (Applied Biosystems, Waltham, MA). The cycle threshold (Ct) of methylated Septin9 was analyzed using 7500 Fast RT-PCR System Software (Applied Biosystems). According to the instructions, when the cycle threshold (Ct) value of ACTB was less than or equal to 33.1, patients with Ct value of mSEPT9 less than or equal to 41.5 were assigned to the mSEPT9 positive group, whereas those with Ct value over 41.5 or not detected were assigned to the mSEPT9 negative group. In case of invalid results when Ct value of ACTB was over 33.1, another test was needed.

### Data and statistic analysis

The data for ColonUSK and the colonoscopy results for all participants were collected and calculated. The sensitivity and specificity were analyzed. Statistical analysis was performed using software SPSS20.0 (SPSS, Chicago, IL).

## Results

### Development, validation, and optimization of the new SEPT 9 assay

The PCR-based Septin9 methylation assay, approved for early detection of colorectal cancer (CRC) in the USA, Europe, and China, measures plasma methylation levels of a CpG-rich fragment located in the Septin9 gene promoter [19]. Although this assay has demonstrated good specificity and compliance, its relatively lower sensitivity for early-stage CRC and precancerous lesions limits its widespread application in CRC screening. Since the approved Septin9 methylation assay detects only one CpG-rich region, we hypothesized that a new assay simultaneously detecting two CpG-rich subregions in CRC could enhance sensitivity, especially for samples with very low abundance of circulating tumor DNA (ctDNA) from patients with early-stage disease. To test this hypothesis, we conducted a Next-Generation Sequencing (NGS) analysis and measured the methylation status of multiple CpG-rich subregions in the Septin9 promoter from CRC tumor tissues, compared with their paired adjacent normal tissues. Two CpG-rich subregions with significantly higher levels of methylation are located within the CpG island at position 151 and were identified (see Supplementary Tables 1 and Table 2 in additional file 1). Subsequently, we developed a new TaqMan probe-based quantitative Methylation-Specific PCR (qMSP) assay for the detection of each of these two CpG-rich subregions in the Septin 9 gene. An internal control targeting the ACTB gene was included in the same reaction of the qMSP assay. The ΔCq value between the target assay and the internal control was used to reflect the relative methylation levels of each target. Additionally, we developed a multiple qMSP assay that simultaneously detects the two CpG-rich subregions using the same color fluorescent-labeled probes. We then compared the performance of the dual qMSP assay with the single qMSP assay using positive and negative control samples, prepared from Hela and Jurkat cell lines. As shown in Table 1, both the dual qMSP assays could detect positive samples with positive detection rates larger than single, but showed either no or extremely low signals for negative controls. Notably, the dual qMSP assay exhibited improved sensitivity with the lowest detection limit of 12 pg/ml, which is higher than that of the single qMSP assay (30 pg/ml).

**Table 1:** Comparison of positive detection rates between the two assays from the training study

Type	Dual-qMSP assay x%(*N*/M)	95% CI	Single-qMSP assay x% (*N*/M)	95% CI
CRC	78.95% (75/95)	69.13-86.37%	77.89% (74/95)	67.99-85.50%
Phase I	73.68% (14/19 )	48.58-89.88%	68.42% (13/19)	43.50-86.44%
Phase II	79% (21/30)	50.44-84.59%	70% (21/30)	50.44-84.59%
Phase III	79.17% (19/24 )	57.29-92.06%	79.17% (19/24)	57.29-92.06%
Phase IV	100% (10/10)	65.55-100.00%	100% (10/10 )	65.55-100.00%
Unclassified	91.67% (11/12 )	59.75-99.56%	91.67% (11/12 )	59.75-99.56%
Advanced adenoma	43.24% (16/37)	27.50-60.36%	27.03% (10/37 )	14.37-44.39%
High-grade intraepithelial neoplasia	65% (13/20)	40.95-83.69%	40% (8/20 )	19.98-63.59%
Villous or diameter ≥ 1 cm	17.65% (3/17)	4.67-44.20%	11.76% (2/17)	2.06-37.75%
Other non-CRC digestive diseases	7.14% (6/84)	84.53-97.06%	7.14% (6/84)	2.96-5.47%

**Table 2:** A summary of enrolled subjects for the opportunity study

Type		Total	Gender		Age			
			Male	Famele	< 50	50–59	60–69	≥ 70
CRC	Total	406	252	154	57	110	137	102
	Phase I	54	30	24	3	17	27	7
	Phase II	133	86	47	14	34	41	44
	Phase III	147	93	54	25	42	53	27
	Phase IV	42	24	18	6	11	11	14
	Unclassified	30	19	11	9	6	5	10
Advanced adenoma	High-grade intraepithelial neoplasia	35	21	14	8	6	14	7
	Villous or diameter ≥ 1 cm	60	37	23	21	19	15	5
Normal		349	170	179	239	68	34	8
Polypus		223	135	88	118	65	25	15
Adenoma		139	86	53	45	48	36	10
Other digestive diseases		79	46	33	46	21	8	4
Other type of tumors		75	44	31	15	21	27	12

### Training test on clinical plasma samples (description of samples used in this study in material and method)

To evaluate the performance of this dual qMSP assay in detecting colorectal cancer (CRC) using plasma samples, we conducted a case-control training study. This involved a comparison of the dual qMSP assay with the single qMSP assay using plasma samples from Renmin Hospital of Wuhan University and the Second People’s Hospital of Shenzhen. Blood samples were collected prior to colonoscopy examination. Following confirmation from colonoscopy and/or pathological diagnosis, the blood samples were categorized for the Septin9 qMSP assays. A total of 216 subjects were recruited for this study and grouped based on their clinical diagnosis, including 95 diagnosed with CRC, 37 with advanced adenomas, and 84 with other types of benign digestive diseases.

The positive detection rate for each group is presented in Table 2. The overall sensitivity for CRC detection was 78.95% with the dual qMSP assay and 77.89% with the single qMSP assay. The difference was primarily observed in phase I cases (73.68% vs. 68.42%). For benign digestive diseases, both assays showed an identical specificity of 92.86% (78/84). Notably, the dual qMSP assay demonstrated a sensitivity of 43.24% for detecting advanced adenomas, which was higher than the 27.03% sensitivity of the single qMSP assay. Particularly, for high-grade intraepithelial neoplasia, the dual assay showed a 65% positive rate, surpassing the 40% rate of the single assay. These results suggest that while detecting dual CpG-rich subregions maintains similar specificity, it enhances the assay’s sensitivity for early-stage CRC and advanced adenoma, especially for high-grade intraepithelial neoplasia in clinical plasma samples.

### Opportunity screening study

**Fig. 1:**
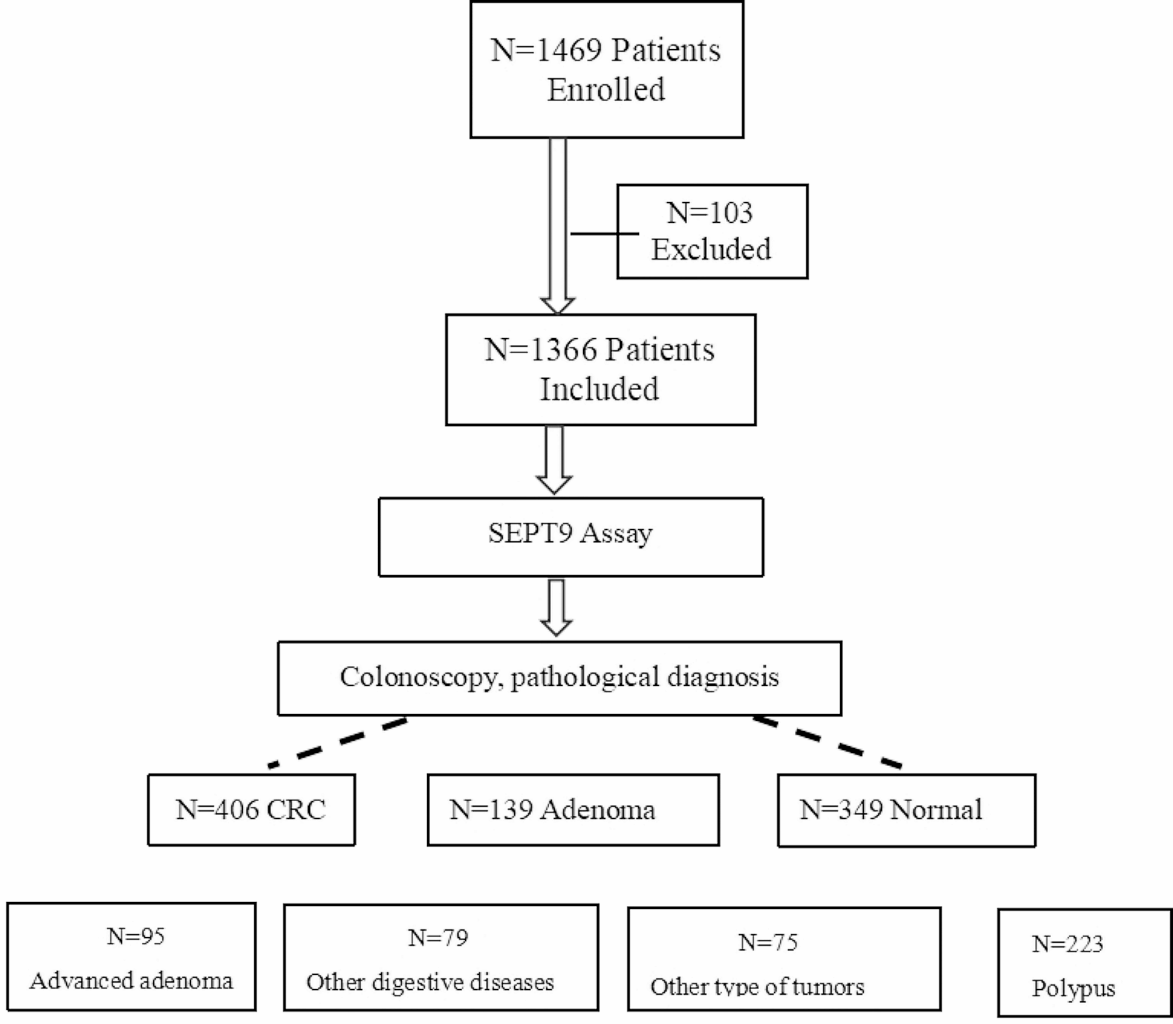
Clinical characteristics of patients and controls

**Fig. 2:**
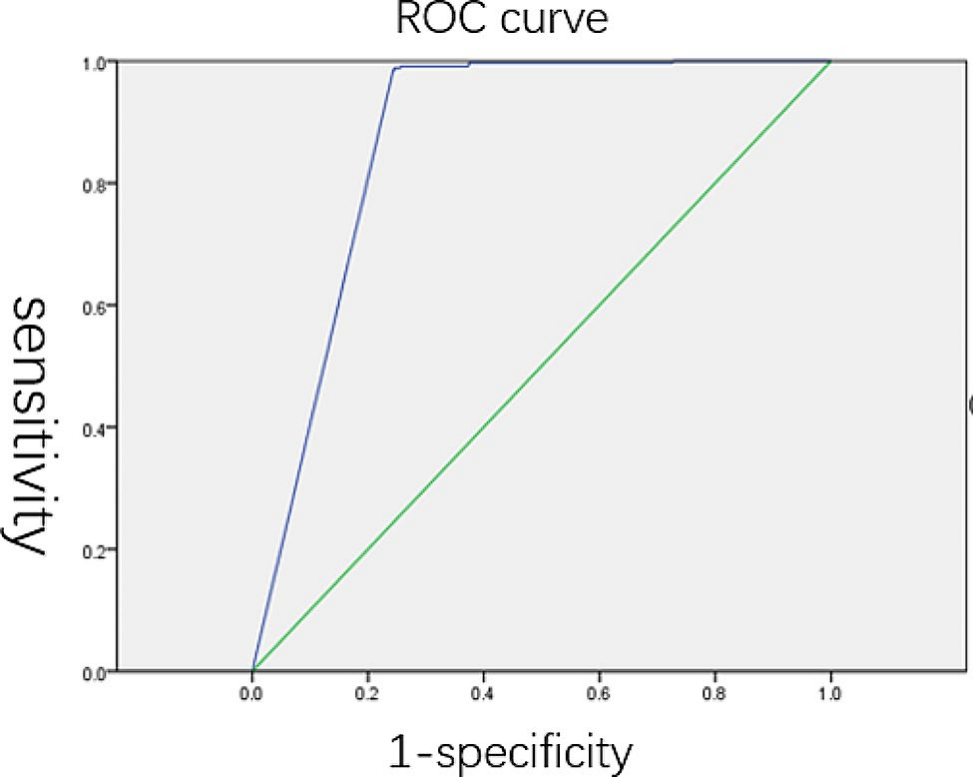
ROC curve for the opportunistic screening trial

To further assess the performance of this dual-qMSP assay in a clinical setting, an opportunistic screening study was designed and conducted across three comprehensive hospitals: Renmin Hospital of Wuhan University, the Second People’s Hospital of Shenzhen, and the Second Affiliated Hospital of Hainan Medical University. The study enrolled subjects from March 2018 until November 2022. As illustrated in Fig. 1 and 469 patients were initially recruited. Of these, 103 cases were excluded due to incomplete information or ineffective results. The remaining 1,366 patients, who had complete information and test results, underwent subsequent colonoscopy and pathology analysis. The cohort comprised 791 males (57.91%) and 575 females (42.09%). The tested subjects included 406 diagnosed with CRC, 95 with advanced adenomas, 139 with adenomas, 223 with polyps, 79 with other types of tumors, 75 with other digestive diseases, and 349 with normal colon findings (Fig. 1, Table 2). The positive detection rates and the specificity for each disease group are shown in Tables 3 and Table 4. The overall clinical sensitivity of the assay for CRC detection was 77.34% (95% CI: 72.89%-81.26%). For advanced adenomas, the clinical sensitivity was 25.26% (95% CI: 17.16%-35.41%). Consistent with the training study, the assay’s clinical sensitivity for detecting high-grade intraepithelial neoplasia was 54.29% (95% CI: 36.87%-70.78%). The overall clinical specificity for CRC was 95.95% (95% CI: 94.36%-97.13%), and for other tumors, it was 88.00% (95% CI: 77.95%-94.03%). The Kappa test value was 0.76, demonstrating a high degree of consistency between the evaluated assay and the clinical ‘gold standard’ in diagnosing CRC. In Fig. 2, there was the ROC (Receiver Operating Characteristic) curve for the opportunistic screening trial. Ct values from 406 CRC patients and 349 No Evidence of Disease (NED) subjects were used to draw the ROC curve. An area under the curve of 0.874 showed both high sensitivity and high specificity of the assay, demonstrating its effective performance in CRC screening.

**Table 3:** Positive testing results for CRC and advanced adenoma from the opportunity screening study

Type	Positive rate	95%CI
CRC	77.34%(314/406)	72.89-81.26%
Phase I	63.11%(36/57)	46.88-73.77%
Phase II	75.19% (100/133)	66.81-82.08%
Phase III	80.27% (118/147)	72.73-86.19%
Phase IV	90.48% (38/42)	76.45-96.90%
Unclassified	81.48% (22/27)	64.55-93.70%
Advanced adenoma	25.26%(24/95)	17.16-35.41%
High-grade intraepithelial neoplasia	54.29% (19/35)	36.87-70.78%
Villous or diameter ≥ 1 cm	8.33% (5/60)	3.11-19.12%
Adenoma		

**Table 4:** Specificity for of the assay from the the opportunity screening study

Type	Negative rate %	95%CI
Non-CRC	95.95% (830/865)	94.36-97.13%
Normal	98.57% (344/349)	96.49-99.47%
Polypus	95.52% (213/223)	91.66-97.71%
Adenoma	93.53% (130/139)	87.71-96.81%
Other digestive diseases	97.47% (77/79)	90.31-99.56%
Other type of tumors	88.00% (66/75)	77.95-94.03%

## Discussion

Our study highlights the potential of a novel Septin9 methylation assay designed for CRC screening, specifically focusing on the simultaneous detection of two different CpG islands of the Septin9 gene in plasma samples. This assay exhibits increased sensitivity in detecting CRC, particularly for high-grade intraepithelial neoplasia. The Kappa test value, a measure of consistency, revealed a high degree of agreement between our assay and the established reference method in diagnosing CRC. The significant proportion of CRC patients observed in our opportunistic screening study aligns closely with that reported in other studies [20, 21], indicating that our findings possess referential value. This finding is significant, as it suggests our assay could substantially enhance the performance of existing plasma Septin9 methylation tests in clinical settings for CRC screening.

In the broader landscape of CRC screening, a variety of methods are currently in use, each presenting unique benefits and drawbacks. Colonoscopy, often regarded as the gold standard, allows for direct visualization of the colon and the ability to perform biopsies or treatment. Despite its effectiveness, colonoscopy has limitations, including the need for invasive bowel preparation and anesthesia. Its accuracy is also contingent on the quality of these preparations and the locations of the lesions. Furthermore, the procedure carries risks of complications and is costlier, requiring specialized medical infrastructure and trained professionals. These factors contribute to lower compliance rates, particularly in developing countries. Fecal occult blood tests (FOBT) offer a non-invasive, cost-effective option for large-scale screening but fall short in terms of sensitivity and specificity. Similarly, stool DNA tests, another non-invasive approach, exhibit high sensitivity for CRC and precancerous lesions but are expensive and do not provide complete colon coverage [22].

Our study’s focus, the blood-based Septin9 gene methylation assay, presents a non-invasive, patient-friendly alternative. This method simplifies the screening process, requiring only a blood sample and avoiding the discomfort associated with other procedures. Despite its advantages, current versions of this assay have shown lower sensitivity compared to stool DNA tests, particularly for early-stage lesions.

The introduction of the Epi proColon, the first FDA-approved blood-based CRC screening assay, marked a significant advancement in this field. This qualitative in vitro Real-Time PCR test detects the methylated SEPT9 gene V2 transcript in CpG island 3 of the promoter region, which is associated with CRC. Recommended for individuals with a normal risk level for CRC who opt out of other screening methods like colonoscopy, the Epi proColon assay is conducted in triplicate and is available in two versions: Epi proColon1.0 and 2.0. The latter version has shown a notable increase in detection sensitivity and specificity, representing a substantial improvement over its predecessor.

Further advancements in this domain include the development of a single-tube methylation specific quantitative PCR assay (mqMSP) by Jin et al. [23]. This assay can analyze plasma samples with minimal tumor DNA content, and in a cohort study, it demonstrated impressive sensitivity of 84.9%, but a moderate specificity of 83.3%. Moreover, it showed superior performance in detecting early-stage CRC and premalignant polyps compared to existing SEPT9 assays. In a separate study involving a longitudinal cohort of CRC patients, the mqMSP assay effectively detected ctDNA in preoperative samples and was indicative of recurrence risk and poorer recurrence-free survival. This study is consistent with our results, and suggest the mqMSP assay is a convenient and economical technology that can cause better routine clinical surveillance of CRC recurrence after surgery.

Another significant development is the novel blood-based gene methylation assay by Fei Xu et al., targeting Septin9, branched-chain amino acid transaminase 1 (BCAT1), and Syndecan-2 (SDC2). This assay was tested on a large cohort and showed promising results in CRC diagnosis, offering high sensitivity of 83.7% and specificity 93.9% [24], pointing out a new direction by clinical examinations of CRC patients. Currently we have also identified a new combination of targets and development of the assay is undergoing.

Our research, alongside the contributions from Jin and Fei Xu et al., underscores the evolving landscape of CRC screening. The development of the blood-based Septin9 methylation assay, the mqMSP assay, and new methylation assay targeting multiple different tumor suppressor genes, heralds a new era in CRC detection. These advancements bring forth more sensitive, specific, and patient-friendly screening options, addressing the limitations of traditional methods. Their implementation in clinical practice holds immense promise for enhancing CRC screening efficiency, improving patient compliance, and ultimately fostering better outcomes in CRC management. This shift towards more sophisticated, non-invasive screening tools is a crucial step in the ongoing effort to combat colorectal cancer effectively.

## Conclusions

In summary, this study showed a novel Septin9 methylation assay (ColonUSK) which designed for CRC screening, specifically focusing on the simultaneous detection of two different CpG islands of the Septin9 gene in plasma samples. This assay exhibits increased sensitivity in detecting CRC, particularly for high-grade intraepithelial neoplasia. We believe that the findings of our study are novel and significant, and the ColonUSK assay could significantly improve CRC screening practices.

### Electronic supplementary material

Below is the link to the electronic supplementary material.

Supplementary Material 1

## Data Availability

Data is provided within the manuscript or supplementary information files.

## Declarations

### Abbreviations

CRC: Colorectal cancer,

FIT: Fecal immunochemical testing,

mtsDNA: multi target stool DNA,

CSCO: Chinese Society of Clinical Oncology,

FOBT: Fecal occult blood testing,

ESD: Post-Endoscopic Submucosal Dissection,

ASIR: Age-standardized incidence rate,

Ct: Cycle threshold,

NGS: Next-Generation Sequencing,

ctDNA: circulating tumor DNA,

qMSP: Quantitative methylation-specific PCR,

BCAT1: Branched-chain amino acid transaminase 1,

SDC2: Syndecan-2,

ROC: Receiver operating characteristic.
