# Supplementary material for: A novel dual-target Septin9 methylation assay for improved detection of early-stage colorectal cancer and high-grade intraepithelial neoplasia
Source: BMC Cancer. 2024 Jul 30;24:916. doi: 10.1186/s12885-024-12645-4 (PMC11290180; doi:10.1186/s12885-024-12645-4)
Supplement: Supplementary file 1 — Supplementary Material 1 [file 12885_2024_12645_MOESM1_ESM.docx]

**Supplementary Table 1. Primer sequences used for NGS**

| assay | primer | 5‘-3’ sequence |
| --- | --- | --- |
| A1 | A1-F | GAGGGGTTGAGGTCGCGT |
|  | A1-R | CCCCTAAACCCCCTAACTCAACTA |
| A2 | A2-F | GTTAGGGGGTTTAGGGGTTTT |
|  | A2-R | ATACCCCTAACAAAATCCC |

**Supplementary Table 2. the methylation rate in different sites for different samples**

There are eight sites selected for PCR detection including 7539377, 7539382, 7539384, 7539386, 7539552, 7539554, 7539556, and 7539561.

| pos |  | Phase 0 | Phase 0 | Phase 0 | Phase 0 | Phase I | Phase I | Phase I | Phase I | Phase I | Phase I | Phase II | Phase II | Phase III | Phase IV | Phase IV |
| --- | --- | --- | --- | --- | --- | --- | --- | --- | --- | --- | --- | --- | --- | --- | --- | --- |
| 7539178 | C | 35.20% | 30.80% | 27.20% | 29.40% | 32.90% | 27.00% | 25.30% | 14.60% | 14.70% | 22.50% | 31.00% | 27.30% | 22.80% | 36.00% | 14.30% |
|  | P | 40.30% | 43.70% | 45.00% | 38.10% | 42.60% | 52.60% | 46.40% | 48.70% | 41.40% | 43.90% | 48.40% | 48.50% | 41.50% | 41.80% | 26.00% |
| 7539181 | C | 34.40% | 39.30% | 28.20% | 34.20% | 29.90% | 26.70% | 29.80% | 29.70% | 22.70% | 17.60% | 31.00% | 41.50% | 41.80% | 26.00% | 48.50% |
|  | P | 31.60% | 26.40% | 29.40% | 35.60% | 49.60% | 29.80% | 32.10% | 34.50% | 27.10% | 19.40% | 36.50% | 25.90% | 48.20% | 35.10% | 40.60% |
| 7539192 | C | 21.20% | 22.20% | 17.90% | 22.30% | 28.60% | 37.40% | 37.40% | 18.80% | 18.80% | 20.50% | 17.70% | 23.80% | 33.30% | 21.00% | 13.10% |
|  | P | 20.00% | 20.60% | 14.20% | 34.20% | 11.20% | 18.60% | 0.40% | 15.30% | 29.50% | 41.10% | 25.50% | 13.50% | 31.20% | 40.80% | 26.90% |
| 7539194 | C | 0.30% | 0.30% | 0.30% | 0.50% | 0.30% | 0.40% | 0.20% | 0.30% | 0.20% | 0.30% | 0.30% | 0.30% | 0.20% | 0.20% | 0.40% |
|  | P | 0.20% | 1.00% | 0.30% | 1.50% | 0.30% | 0.20% | 0.20% | 0.30% | 0.20% | 0.20% | 0.30% | 0.10% | 0.50% | 0.20% | 0.20% |
| 7539203 | C | 0.20% | 0.20% | 0.30% | 0.40% | 0.50% | 0.40% | 0.20% | 0.30% | 0.30% | 0.10% | 0.20% | 0.10% | 0.20% | 0.20% | 0.30% |
|  | P | 0.30% | 0.30% | 0.30% | 0.30% | 0.30% | 0.20% | 0.30% | 0.10% | 0.50% | 0.50% | 0.60% | 0.60% | 0.30% | 0.20% | 0.20% |
| 7539218 | C | 0.40% | 0.65% | 0.25% | 0.31% | 0.34% | 0.21% | 0.28% | 0.29% | 0.64% | 0.48% | 0.35% | 0.45% | 0.65% | 0.21% | 0.35% |
|  | P | 0.65% | 0.45% | 0.51% | 0.53% | 0.24% | 0.35% | 0.25% | 0.16% | 0.35% | 0.21% | 0.25% | 0.24% | 0.35% | 0.15% | 0.36% |
| 7539223 | C | 0.62% | 0.53% | 0.46% | 0.42% | 0.65% | 0.15% | 0.42% | 0.53% | 0.65% | 0.48% | 0.46% | 0.53% | 0.51% | 0.52% | 0.43% |
|  | P | 0.59% | 0.42% | 0.35% | 0.45% | 0.46% | 0.35% | 0.48% | 0.46% | 0.65% | 0.42% | 0.43% | 0.45% | 0.15% | 0.15% | 0.16% |
| 7539227 | C | 0.45% | 0.35% | 0.48% | 0.46% | 0.35% | 0.65% | 0.46% | 0.49% | 0.52% | 0.51% | 0.62% | 0.46% | 0.62% | 0.42% | 0.63% |
|  | P | 0.42% | 0.35% | 0.65% | 0.46% | 0.46% | 0.15% | 0.64% | 0.82% | 0.48% | 0.63% | 0.75% | 0.43% | 0.45% | 0.35% | 0.48% |
| 7539251 | C | 0.58% | 0.64% | 0.35% | 0.48% | 0.58% | 0.65% | 0.46% | 0.75% | 0.63% | 0.65% | 0.75% | 0.76% | 0.84% | 0.96% | 0.61% |
|  | P | 0.43% | 0.52% | 0.46% | 0.48% | 0.42% | 0.62% | 0.43% | 0.35% | 0.42% | 0.63% | 0.85% | 0.64% | 0.64% | 0.35% | 0.34% |
| 7539259 | C | 0.75% | 0.65% | 0.35% | 0.46% | 0.35% | 0.85% | 0.64% | 0.35% | 0.64% | 0.65% | 0.18% | 0.65% | 0.53% | 0.41% | 0.46% |
|  | P | 0.65% | 0.42% | 0.65% | 0.48% | 0.43% | 0.35% | 0.54% | 0.62% | 0.34% | 0.42% | 0.15% | 0.16% | 0.62% | 0.51% | 0.62% |
| 7539293 | C | 0.40% | 0.25% | 0.35% | 0.64% | 0.18% | 0.49% | 0.47% | 0.46% | 0.43% | 0.41% | 0.47% | 0.49% | 0.42% | 0.48% | 0.41% |
|  | P | 0.35% | 0.34% | 0.39% | 0.37% | 0.38% | 0.34% | 0.34% | 0.36% | 0.15% | 0.16% | 0.34% | 0.35% | 0.31% | 0.35% | 0.39% |
| 7539297 | C | 0.58% | 0.59% | 0.54% | 0.64% | 0.61% | 0.34% | 0.85% | 0.49% | 0.74% | 0.64% | 0.85% | 0.84% | 0.97% | 0.49% | 0.48% |
|  | P | 0.47% | 0.49% | 0.46% | 0.46% | 0.48% | 0.47% | 0.46% | 0.43% | 0.86% | 0.49% | 0.47% | 0.46% | 0.35% | 0.48% | 0.35% |
| 7539311 | C | 0.95% | 0.76% | 0.84% | 0.64% | 0.84% | 0.94% | 0.76% | 0.85% | 0.85% | 0.94% | 0.64% | 0.95% | 0.97% | 0.96% | 0.98% |
|  | P | 0.95% | 0.96% | 0.74% | 0.70% | 0.46% | 0.48% | 0.46% | 0.47% | 0.64% | 0.48% | 0.48% | 0.62% | 0.64% | 0.47% | 0.94% |
| 7539325 | C | 0.65% | 0.84% | 0.94% | 0.97% | 0.64% | 0.85% | 0.87% | 0.62% | 0.34% | 0.38% | 0.75% | 0.71% | 0.72% | 0.76% | 0.78% |
|  | P | 0.42% | 0.43% | 0.47% | 0.48% | 0.41% | 0.43% | 0.29% | 0.27% | 0.28% | 0.35% | 0.31% | 0.36% | 0.35% | 0.38% | 0.31% |
| 7539330 | C | 1.21% | 1.32% | 1.58% | 1.35% | 1.45% | 1.36% | 1.25% | 1.58% | 2.12% | 2.16% | 2.48% | 2.65% | 3.12% | 3.48% | 3.85% |
|  | P | 0.25% | 0.35% | 0.85% | 0.67% | 0.95% | 0.67% | 0.47% | 0.37% | 0.74% | 0.85% | 0.72% | 0.73% | 0.92% | 0.46% | 0.46% |
| 7539336 | C | 2.24% | 2.36% | 3.25% | 3.46% | 3.78% | 3.49% | 3.68% | 3.18% | 3.65% | 3.47% | 3.15% | 3.69% | 3.15% | 3.47% | 3.69% |
|  | P | 2.81% | 2.69% | 2.48% | 2.46% | 1.58% | 1.64% | 1.69% | 1.85% | 1.46% | 1.65% | 1.58% | 1.47% | 1.69% | 1.69% | 1.47% |
| 7539349 | C | 2.64% | 2.64% | 2.48% | 2.96% | 2.74% | 2.68% | 3.45% | 3.69% | 4.12% | 4.59% | 4.96% | 4.75% | 4.63% | 7.15% | 8.12% |
|  | P | 0.15% | 0.75% | 0.65% | 0.45% | 0.46% | 0.58% | 0.58% | 0.68% | 0.12% | 0.35% | 0.17% | 0.16% | 1.12% | 1.58% | 2.12% |
| 7539353 | C | 8.59% | 8.64% | 8.14% | 6.54% | 4.56% | 8.45% | 1.12% | 4.25% | 6.25% | 7.45% | 4.12% | 6.56% | 5.58% | 2.52% | 3.45% |
|  | P | 2.59% | 2.64% | 3.15% | 3.85% | 3.46% | 3.45% | 2.13% | 3.45% | 3.48% | 3.47% | 3.69% | 3.58% | 2.12% | 2.47% | 2.64% |
| 7539360 | C | 3.25% | 4.15% | 3.16% | 2.16% | 2.14% | 2.43% | 3.25% | 2.58% | 2.15% | 2.14% | 2.64% | 2.58% | 2.78% | 2.34% | 2.15% |
|  | P | 1.24% | 1.34% | 1.25% | 0.25% | 0.34% | 0.25% | 0.35% | 0.14% | 0.74% | 0.34% | 0.58% | 0.34% | 0.85% | 0.35% | 0.25% |
| 7539366 | C | 2.64% | 2.85% | 3.14% | 3.65% | 3.47% | 3.58% | 3.14% | 3.16% | 3.74% | 3.25% | 3.25% | 3.18% | 3.78% | 3.45% | 3.25% |
|  | P | 0.68% | 0.64% | 0.87% | 0.28% | 0.47% | 0.67% | 0.98% | 0.48% | 0.64% | 0.85% | 0.25% | 0.34% | 0.85% | 0.64% | 0.51% |
| 7539371 | C | 3.46% | 2.15% | 2.48% | 2.46% | 1.35% | 1.58% | 1.35% | 1.48% | 1.97% | 1.64% | 1.47% | 1.35% | 1.39% | 1.75% | 1.58% |
|  | P | 1.24% | 0.25% | 1.57% | 1.32% | 0.64% | 0.48% | 0.89% | 0.97% | 0.64% | 0.75% | 0.46% | 0.38% | 0.85% | 0.18% | 0.74% |
| 7539377 | C | 7.80% | 7.40% | 7.30% | 7.80% | 7.30% | 7.40% | 8.20% | 10.20% | 6.40% | 5.20% | 11.20% | 12.30% | 21.20% | 16.50% | 14.20% |
|  | P | 0.20% | 0.40% | 1.30% | 2.10% | 1.20% | 1.30% | 1.50% | 2.10% | 2.20% | 2.50% | 2.10% | 3.20% | 10.20% | 11.50% | 16.20% |
| 7539382 | C | 18.10% | 19.60% | 15.80% | 18.70% | 35.60% | 21.60% | 27.50% | 31.40% | 19.50% | 32.70% | 29.30% | 35.60% | 41.00% | 42.01% | 51.42% |
|  | P | 1.60% | 0.70% | 0.20% | 1.60% | 1.30% | 1.90% | 1.80% | 2.20% | 1.10% | 2.00% | 2.70% | 3.40% | 25.10% | 14.40% | 14.30% |
| 7539384 | C | 7.40% | 7.10% | 6.20% | 7.10% | 3.50% | 13.10% | 13.20% | 23.70% | 22.40% | 21.60% | 32.80% | 52.90% | 51.10% | 40.90% | 38.60% |
|  | P | 1.20% | 1.30% | 0.50% | 0.80% | 1.60% | 1.90% | 2.60% | 3.60% | 1.80% | 3.60% | 19.60% | 15.80% | 41.00% | 35.60% | 21.60% |
| 7539386 | C | 12.00% | 14.10% | 12.00% | 6.90% | 13.90% | 10.20% | 8.30% | 6.50% | 3.50% | 13.80% | 23.40% | 21.60% | 27.50% | 31.40% | 19.50% |
|  | P | 1.40% | 1.60% | 0.70% | 0.20% | 1.60% | 1.60% | 2.10% | 1.50% | 3.10% | 0.10% | 0.80% | 18.80% | 18.80% | 20.50% | 17.70% |
| 7539397 | C | 3.21% | 2.50% | 2.10% | 1.85% | 1.26% | 1.10% | 1.12% | 1.35% | 3.21% | 3.54% | 10.21% | 5.65% | 5.11% | 4.54% | 5.11% |
|  | P | 1.25% | 1.65% | 1.30% | 2.10% | 1.20% | 1.30% | 1.50% | 2.10% | 2.20% | 2.50% | 2.10% | 3.20% | 10.20% | 2.52% | 6.21% |
| 7539400 | C | 4.21% | 3.21% | 3.28% | 3.21% | 3.25% | 3.64% | 3.78% | 3.95% | 3.48% | 3.76% | 3.46% | 3.85% | 8.25% | 7.65% | 9.15% |
|  | P | 1.40% | 0.80% | 0.40% | 1.46% | 1.64% | 1.54% | 1.80% | 2.20% | 1.20% | 3.10% | 3.20% | 2.10% | 12.20% | 13.20% | 11.20% |
| 7539404 | C | 2.62% | 3.15% | 3.58% | 3.46% | 3.12% | 4.21% | 4.32% | 5.20% | 3.25% | 3.12% | 10.20% | 9.25% | 6.52% | 12.20% | 13.20% |
|  | P | 2.20% | 2.32% | 3.13% | 2.32% | 3.11% | 2.52% | 3.12% | 3.60% | 2.23% | 3.60% | 11.21% | 8.21% | 6.25% | 10.21% | 13.22% |
| 7539455 | C | 3.25% | 3.65% | 4.12% | 4.52% | 5.62% | 4.25% | 3.58% | 4.21% | 5.12% | 13.80% | 13.25% | 14.52% | 10.25% | 10.25% | 11.85% |
|  | P | 2.12% | 1.60% | 3.12% | 3.85% | 3.25% | 3.11% | 2.10% | 3.12% | 3.10% | 3.25% | 2.12% | 12.54% | 9.52% | 10.31% | 11.21% |
| 7539458 | C | 3.21% | 2.46% | 1.46% | 3.21% | 1.45% | 2.35% | 2.54% | 1.25% | 1.64% | 2.13% | 2.45% | 2.34% | 3.25% | 2.15% | 2.11% |
|  | P | 2.13% | 2.25% | 1.32% | 1.24% | 0.95% | 1.45% | 2.15% | 1.45% | 1.27% | 1.58% | 1.46% | 1.52% | 2.12% | 1.54% | 1.35% |
| 7539481 | C | 2.25% | 2.13% | 3.14% | 2.15% | 1.98% | 1.45% | 3.21% | 1.25% | 2.45% | 2.21% | 2.20% | 1.98% | 1.24% | 1.45% | 1.62% |
|  | P | 1.12% | 1.45% | 2.32% | 2.45% | 1.45% | 1.65% | 1.12% | 1.45% | 2.12% | 2.12% | 2.65% | 1.54% | 1.01% | 1.02% | 1.01% |
| 7539483 | C | 3.12% | 2.25% | 4.54% | 3.12% | 2.45% | 2.12% | 3.21% | 3.45% | 2.52% | 3.21% | 3.45% | 3.21% | 2.25% | 2.45% | 2.47% |
|  | P | 1.21% | 2.25% | 2.65% | 3.45% | 1.54% | 1.25% | 1.35% | 1.45% | 1.25% | 0.35% | 0.95% | 2.45% | 6.54% | 4.45% | 2.25% |
| 7539487 | C | 3.22% | 4.12% | 3.85% | 3.46% | 3.11% | 2.85% | 2.46% | 3.12% | 3.22% | 3.14% | 3.05% | 3.65% | 2.52% | 3.12% | 3.65% |
|  | P | 1.65% | 1.58% | 1.46% | 2.54% | 2.65% | 2.45% | 2.12% | 2.13% | 1.45% | 1.25% | 2.13% | 2.65% | 2.15% | 2.46% | 1.25% |
| 7539489 | C | 4.05% | 4.50% | 4.32% | 3.48% | 3.64% | 2.45% | 2.85% | 3.45% | 2.65% | 2.46% | 2.45% | 2.12% | 2.32% | 2.14% | 2.25% |
|  | P | 1.25% | 2.32% | 2.45% | 2.47% | 1.58% | 1.65% | 1.95% | 1.32% | 1.75% | 2.45% | 1.32% | 1.58% | 1.20% | 1.85% | 1.65% |
| 7539492 | C | 3.65% | 3.45% | 3.58% | 3.95% | 3.65% | 2.65% | 2.75% | 2.35% | 2.45% | 2.65% | 2.12% | 2.35% | 2.14% | 2.13% | 2.47% |
|  | P | 2.12% | 2.32% | 2.12% | 2.44% | 1.25% | 1.02% | 1.35% | 1.68% | 1.95% | 1.75% | 1.65% | 1.55% | 1.02% | 1.32% | 1.02% |
| 7539504 | C | 2.85% | 2.64% | 2.89% | 3.12% | 3.45% | 3.25% | 2.65% | 2.45% | 2.85% | 2.32% | 1.95% | 1.45% | 3.45% | 3.65% | 4.01% |
|  | P | 2.01% | 2.32% | 1.45% | 1.95% | 1.54% | 1.65% | 2.02% | 2.35% | 2.12% | 2.12% | 2.12% | 1.65% | 1.02% | 1.32% | 1.25% |
| 7539506 | C | 3.25% | 2.12% | 2.35% | 2.45% | 3.25% | 3.65% | 2.45% | 2.12% | 2.68% | 3.12% | 3.12% | 2.45% | 2.25% | 2.12% | 2.32% |
|  | P | 0.95% | 0.96% | 1.12% | 1.11% | 1.32% | 1.58% | 1.32% | 1.58% | 1.54% | 1.98% | 2.01% | 2.35% | 1.54% | 2.58% | 2.65% |
| 7539515 | C | 3.58% | 3.69% | 3.78% | 4.15% | 5.16% | 6.54% | 7.55% | 7.15% | 5.12% | 8.12% | 9.45% | 9.99% | 9.65% | 6.56% | 6.32% |
|  | P | 2.65% | 2.35% | 2.46% | 2.46% | 1.25% | 4.52% | 5.65% | 6.65% | 4.12% | 3.65% | 3.45% | 3.45% | 4.58% | 5.15% | 6.12% |
| 7539522 | C | 3.12% | 2.15% | 2.12% | 3.65% | 2.85% | 2.12% | 2.68% | 3.12% | 3.32% | 2.14% | 2.25% | 2.12% | 2.85% | 3.12% | 4.12% |
|  | P | 1.25% | 1.35% | 1.65% | 1.58% | 1.75% | 1.46% | 1.59% | 2.45% | 1.45% | 1.65% | 1.48% | 1.47% | 1.89% | 1.65% | 1.48% |
| 7539525 | C | 3.12% | 4.65% | 3.25% | 3.46% | 2.15% | 2.48% | 2.98% | 2.47% | 2.63% | 2.48% | 2.16% | 2.45% | 2.65% | 2.13% | 2.18% |
|  | P | 1.68% | 2.02% | 2.35% | 2.45% | 2.65% | 2.32% | 2.45% | 1.48% | 1.65% | 1.48% | 1.63% | 1.47% | 1.85% | 1.65% | 1.65% |
| 7539531 | C | 2.65% | 2.48% | 2.64% | 2.63% | 3.12% | 3.25% | 4.50% | 6.25% | 7.45% | 6.46% | 4.15% | 4.35% | 3.25% | 3.65% | 3.25% |
|  | P | 2.12% | 2.65% | 2.45% | 2.69% | 2.48% | 2.75% | 2.65% | 2.64% | 2.35% | 2.48% | 2.68% | 2.67% | 2.46% | 2.85% | 2.46% |
| 7539539 | C | 2.13% | 3.15% | 3.45% | 3.25% | 4.12% | 2.58% | 3.13% | 3.65% | 2.45% | 2.46% | 2.18% | 2.46% | 2.62% | 2.16% | 2.65% |
|  | P | 1.65% | 1.85% | 1.64% | 1.75% | 1.64% | 1.96% | 1.36% | 1.42% | 1.63% | 1.25% | 2.15% | 1.98% | 2.95% | 2.94% | 2.94% |
| 7539542 | C | 3.65% | 3.25% | 3.25% | 4.15% | 4.16% | 4.18% | 4.65% | 4.78% | 4.25% | 4.36% | 3.45% | 4.58% | 4.25% | 4.16% | 4.15% |
|  | P | 3.25% | 3.65% | 3.45% | 3.65% | 3.12% | 6.68% | 6.58% | 5.15% | 2.15% | 2.32% | 3.45% | 3.65% | 5.12% | 5.15% | 6.12% |
| 7539552 | C | 8.25% | 9.58% | 9.65% | 10.12% | 10.45% | 10.68% | 10.75% | 10.75% | 11.25% | 16.25% | 16.84% | 20.12% | 20.46% | 25.64% | 26.12% |
|  | P | 0.25% | 0.34% | 0.85% | 0.56% | 2.45% | 2.32% | 1.35% | 1.48% | 1.46% | 1.47% | 2.12% | 2.31% | 3.15% | 3.46% | 3.14% |
| 7539554 | C | 9.25% | 9.64% | 9.34% | 9.76% | 10.20% | 12.13% | 14.16% | 12.52% | 13.25% | 14.16% | 15.64% | 16.45% | 25.46% | 26.12% | 28.12% |
|  | P | 0.14% | 0.41% | 0.34% | 0.78% | 0.64% | 1.25% | 1.48% | 1.64% | 1.47% | 1.64% | 2.65% | 2.47% | 2.65% | 2.47% | 3.45% |
| 7539556 | C | 9.68% | 10.25% | 12.45% | 13.56% | 15.45% | 12.46% | 15.12% | 16.32% | 17.45% | 18.12% | 20.12% | 25.46% | 20.12% | 24.45% | 26.22% |
|  | P | 1.25% | 2.13% | 2.45% | 3.15% | 2.14% | 1.48% | 1.47% | 2.34% | 3.64% | 2.45% | 2.12% | 2.16% | 3.14% | 3.12% | 3.25% |
| 7539561 | C | 10.25% | 11.24% | 11.64% | 11.87% | 11.34% | 12.45% | 20.14% | 20.64% | 21.25% | 22.65% | 25.14% | 30.12% | 30.45% | 30.98% | 30.46% |
|  | P | 0.15% | 0.45% | 0.64% | 0.12% | 1.25% | 1.34% | 1.25% | 1.47% | 1.68% | 1.47% | 2.12% | 2.34% | 2.15% | 4.32% | 4.97% |
| 7539583 | C | 5.21% | 5.46% | 4.25% | 6.21% | 4.21% | 3.62% | 3.58% | 4.12% | 2.16% | 2.13% | 2.41% | 3.32% | 3.31% | 2.15% | 2.45% |
|  | P | 2.12% | 1.15% | 1.32% | 1.78% | 1.98% | 1.85% | 1.24% | 1.16% | 1.45% | 0.45% | 0.15% | 1.65% | 1.48% | 1.01% | 1.03% |
| 7539590 | C | 6.54% | 4.56% | 3.65% | 3.78% | 4.51% | 3.24% | 4.18% | 4.58% | 4.74% | 4.28% | 5.28% | 6.18% | 12.13% | 12.47% | 12.64% |
|  | P | 1.24% | 1.35% | 1.11% | 0.98% | 1.34% | 0.78% | 0.64% | 1.24% | 1.27% | 2.12% | 2.34% | 2.52% | 2.46% | 4.50% | 4.65% |
| 7539592 | C | 3.25% | 1.02% | 1.34% | 1.78% | 1.64% | 1.25% | 1.26% | 1.34% | 1.24% | 1.47% | 1.64% | 1.65% | 1.25% | 1.75% | 1.67% |
|  | P | 0.25% | 0.34% | 0.35% | 0.25% | 0.14% | 0.34% | 0.25% | 0.46% | 0.15% | 0.15% | 0.16% | 0.64% | 0.48% | 0.48% | 0.19% |
| 7539599 | C | 1.85% | 1.97% | 1.36% | 1.74% | 1.26% | 1.74% | 1.64% | 1.25% | 1.74% | 1.64% | 1.25% | 1.75% | 1.34% | 1.74% | 1.25% |
|  | P | 0.47% | 0.16% | 1.85% | 1.46% | 0.52% | 0.43% | 0.52% | 0.47% | 0.64% | 0.34% | 0.58% | 0.18% | 0.64% | 0.34% | 0.15% |
| 7539601 | C | 1.45% | 1.34% | 1.25% | 1.57% | 1.47% | 1.34% | 1.98% | 1.24% | 1.26% | 1.28% | 1.24% | 1.26% | 1.27% | 1.58% | 1.34% |
|  | P | 0.84% | 0.64% | 0.34% | 0.25% | 0.45% | 0.64% | 0.34% | 0.25% | 0.85% | 0.35% | 0.25% | 0.25% | 0.42% | 0.43% | 0.18% |
| 7539622 | C | 1.28% | 1.41% | 1.43% | 1.48% | 1.49% | 1.47% | 1.25% | 1.78% | 1.72% | 1.79% | 1.35% | 1.34% | 1.25% | 1.85% | 1.64% |
|  | P | 0.34% | 0.25% | 0.64% | 0.62% | 0.28% | 0.34% | 0.34% | 0.26% | 0.18% | 0.47% | 0.64% | 0.48% | 0.64% | 0.34% | 0.58% |
| 7539629 | C | 1.58% | 1.52% | 1.53% | 1.54% | 1.56% | 1.54% | 1.74% | 1.94% | 1.84% | 1.34% | 1.34% | 1.25% | 1.42% | 1.48% | 1.52% |
|  | P | 0.64% | 0.58% | 0.47% | 0.34% | 0.34% | 0.16% | 0.48% | 0.16% | 0.46% | 0.78% | 0.76% | 0.46% | 0.84% | 0.94% | 0.43% |
| 7539656 | C | 1.34% | 1.36% | 1.38% | 1.39% | 1.95% | 1.94% | 1.64% | 1.58% | 1.64% | 1.57% | 1.25% | 1.21% | 1.26% | 1.34% | 1.35% |
|  | P | 0.64% | 0.48% | 0.28% | 0.64% | 0.18% | 0.46% | 0.69% | 0.75% | 0.16% | 0.34% | 0.45% | 0.48% | 0.48% | 0.19% | 0.64% |
| 7539660 | C | 2.25% | 2.24% | 2.16% | 2.28% | 2.41% | 1.25% | 1.69% | 1.74% | 1.67% | 1.58% | 1.69% | 1.58% | 1.97% | 1.67% | 2.12% |
|  | P | 0.48% | 0.34% | 0.35% | 0.15% | 0.16% | 0.75% | 0.64% | 0.15% | 0.94% | 0.48% | 0.16% | 0.34% | 0.16% | 0.18% | 0.64% |
| 7539662 | C | 1.58% | 1.67% | 1.94% | 1.58% | 1.64% | 1.58% | 1.64% | 1.25% | 1.74% | 1.64% | 1.58% | 1.58% | 1.52% | 1.64% | 1.68% |
|  | P | 0.85% | 0.64% | 0.34% | 0.85% | 0.46% | 0.29% | 0.75% | 0.46% | 0.25% | 0.64% | 0.16% | 0.16% | 0.16% | 0.18% | 0.46% |
| 7539666 | C | 1.42% | 1.46% | 1.48% | 1.47% | 1.95% | 1.64% | 1.25% | 1.64% | 1.48% | 1.75% | 1.64% | 1.35% | 1.58% | 1.52% | 1.54% |
|  | P | 0.64% | 0.25% | 0.34% | 0.47% | 0.46% | 0.18% | 0.31% | 0.68% | 0.18% | 0.85% | 0.95% | 0.64% | 0.58% | 0.25% | 0.64% |
| 7539685 | C | 1.24% | 1.34% | 1.84% | 1.74% | 1.32% | 1.25% | 1.34% | 1.24% | 1.58% | 1.67% | 1.69% | 1.49% | 1.34% | 1.34% | 1.20% |
|  | P | 0.94% | 0.24% | 0.34% | 0.58% | 0.54% | 0.34% | 0.15% | 0.17% | 0.16% | 0.47% | 0.34% | 0.38% | 0.48% | 0.46% | 0.48% |
| 7539698 | C | 1.41% | 1.42% | 1.43% | 1.45% | 1.95% | 1.64% | 1.34% | 1.24% | 1.35% | 1.64% | 1.45% | 1.54% | 1.35% | 1.25% | 1.34% |
|  | P | 0.12% | 0.25% | 0.28% | 0.34% | 0.47% | 0.64% | 0.48% | 0.35% | 0.48% | 0.47% | 0.64% | 0.35% | 0.25% | 0.25% | 0.15% |
| 7539700 | C | 1.84% | 1.24% | 1.47% | 1.34% | 1.85% | 1.25% | 1.34% | 1.36% | 1.47% | 1.64% | 1.35% | 1.25% | 1.35% | 1.41% | 1.36% |
|  | P | 0.51% | 0.52% | 0.58% | 0.75% | 0.53% | 0.41% | 0.43% | 0.59% | 0.47% | 0.53% | 0.53% | 0.52% | 0.41% | 0.47% | 0.48% |
| 7539705 | C | 1.74% | 1.94% | 1.35% | 1.34% | 1.14% | 1.15% | 1.35% | 1.35% | 1.46% | 1.58% | 1.34% | 1.34% | 1.35% | 1.25% | 1.25% |
|  | P | 0.58% | 0.74% | 0.64% | 0.35% | 0.15% | 0.42% | 0.36% | 0.69% | 0.35% | 0.41% | 0.42% | 0.74% | 0.34% | 0.25% | 0.14% |
| 7539713 | C | 1.47% | 1.35% | 1.25% | 1.34% | 1.28% | 1.41% | 1.34% | 1.35% | 1.25% | 1.26% | 1.34% | 1.25% | 1.24% | 1.88% | 1.78% |
|  | P | 0.58% | 0.35% | 0.25% | 0.64% | 0.58% | 0.35% | 0.58% | 0.47% | 0.34% | 0.28% | 0.21% | 0.38% | 0.31% | 0.57% | 0.47% |
| 7539726 | C | 1.34% | 1.36% | 1.38% | 1.47% | 1.34% | 1.58% | 1.47% | 1.34% | 1.25% | 1.36% | 1.25% | 1.41% | 1.35% | 1.38% | 1.39% |
|  | P | 0.13% | 0.25% | 0.42% | 0.34% | 0.25% | 0.34% | 0.39% | 0.47% | 0.25% | 0.41% | 0.43% | 0.58% | 0.25% | 0.41% | 0.43% |
| 7539734 | C | 1.68% | 1.97% | 1.48% | 1.47% | 1.25% | 1.14% | 1.34% | 1.78% | 1.46% | 1.25% | 1.72% | 1.76% | 1.95% | 1.64% | 1.25% |
|  | P | 0.58% | 0.59% | 0.46% | 0.25% | 0.47% | 0.58% | 0.34% | 0.74% | 0.15% | 0.35% | 0.42% | 0.35% | 0.85% | 0.74% | 0.34% |
| 7539747 | C | 1.25% | 1.42% | 1.41% | 1.34% | 1.36% | 1.48% | 1.34% | 1.25% | 1.58% | 1.46% | 1.47% | 1.35% | 1.25% | 1.78% | 1.34% |
|  | P | 0.58% | 0.34% | 0.26% | 0.14% | 0.35% | 0.25% | 0.85% | 0.34% | 0.15% | 0.17% | 0.34% | 0.25% | 0.17% | 0.25% | 0.16% |
| 7539765 | C | 1.25% | 1.34% | 1.25% | 1.85% | 1.64% | 1.25% | 1.47% | 1.35% | 1.36% | 1.47% | 1.25% | 1.35% | 1.47% | 1.64% | 1.25% |
|  | P | 0.25% | 0.34% | 0.25% | 0.15% | 0.85% | 0.74% | 0.46% | 0.48% | 0.75% | 0.64% | 0.15% | 0.16% | 0.47% | 0.48% | 0.34% |
| 7539773 | C | 1.48% | 1.34% | 1.25% | 1.64% | 1.28% | 1.34% | 1.25% | 1.84% | 1.64% | 1.75% | 1.35% | 1.28% | 1.34% | 1.26% | 1.47% |
|  | P | 0.15% | 0.17% | 0.47% | 0.34% | 0.58% | 0.18% | 0.49% | 0.48% | 0.47% | 0.16% | 0.47% | 0.18% | 0.14% | 0.16% | 0.18% |
| 7539782 | C | 1.85% | 1.64% | 1.47% | 1.34% | 1.25% | 1.34% | 1.64% | 1.68% | 1.69% | 1.67% | 1.35% | 1.34% | 1.21% | 1.23% | 1.24% |
|  | P | 0.84% | 0.87% | 0.97% | 0.47% | 0.35% | 0.15% | 0.34% | 0.14% | 0.61% | 0.25% | 0.34% | 0.18% | 0.14% | 0.34% | 0.35% |
| 7539785 | C | 1.28% | 1.24% | 1.34% | 1.28% | 1.95% | 1.48% | 1.64% | 1.47% | 1.34% | 1.24% | 1.51% | 1.29% | 1.54% | 1.26% | 1.28% |
|  | P | 0.58% | 0.25% | 0.16% | 0.35% | 0.25% | 0.14% | 0.34% | 0.25% | 0.25% | 0.67% | 0.47% | 0.64% | 0.25% | 0.35% | 0.47% |
| 7539787 | C | 1.02% | 1.24% | 1.35% | 1.58% | 1.34% | 1.47% | 1.39% | 1.45% | 1.36% | 1.47% | 1.34% | 1.21% | 1.32% | 1.35% | 1.34% |
|  | P | 0.58% | 0.34% | 0.35% | 0.16% | 0.25% | 0.34% | 0.35% | 0.18% | 0.46% | 0.48% | 0.46% | 0.15% | 0.15% | 0.16% | 0.47% |
| 7539789 | C | 0.64% | 0.35% | 0.25% | 0.34% | 0.28% | 0.34% | 0.34% | 0.36% | 0.28% | 0.14% | 0.64% | 0.85% | 0.34% | 0.16% | 0.41% |
|  | P | 0.47% | 0.34% | 0.25% | 0.46% | 0.34% | 0.28% | 0.34% | 0.41% | 0.16% | 0.35% | 0.15% | 0.35% | 0.28% | 0.46% | 0.35% |
| 7539795 | C | 0.15% | 0.34% | 0.64% | 0.25% | 0.85% | 0.46% | 0.34% | 0.25% | 0.46% | 0.18% | 0.34% | 0.18% | 0.15% | 0.34% | 0.34% |
|  | P | 0.48% | 0.14% | 0.34% | 0.18% | 0.64% | 0.15% | 0.18% | 0.34% | 0.18% | 0.34% | 0.34% | 0.36% | 0.18% | 0.64% | 0.34% |
| 7539801 | C | 0.25% | 0.25% | 0.16% | 0.35% | 0.15% | 0.15% | 0.14% | 0.35% | 0.16% | 0.35% | 0.34% | 0.25% | 0.34% | 0.31% | 0.28% |
|  | P | 0.25% | 0.16% | 0.35% | 0.15% | 0.34% | 0.34% | 0.38% | 0.16% | 0.35% | 0.16% | 0.25% | 0.16% | 0.25% | 0.16% | 0.14% |
| 7539807 | C | 0.18% | 0.34% | 0.25% | 0.16% | 0.34% | 0.34% | 0.15% | 0.35% | 0.34% | 0.17% | 0.61% | 0.34% | 0.34% | 0.36% | 0.19% |
|  | P | 0.85% | 0.61% | 0.43% | 0.46% | 0.16% | 0.35% | 0.35% | 0.14% | 0.16% | 0.34% | 0.16% | 0.34% | 0.35% | 0.35% | 0.35% |
| 7539809 | C | 0.84% | 0.64% | 0.34% | 0.30% | 0.47% | 0.34% | 0.24% | 0.31% | 0.31% | 0.15% | 0.34% | 0.16% | 0.18% | 0.16% | 0.21% |
|  | P | 0.64% | 0.34% | 0.15% | 0.35% | 0.16% | 0.18% | 0.17% | 0.13% | 0.46% | 0.35% | 0.26% | 0.85% | 0.94% | 0.64% | 0.64% |
| 7539818 | C | 0.64% | 0.35% | 0.15% | 0.18% | 0.34% | 0.32% | 0.25% | 0.16% | 0.17% | 0.18% | 0.16% | 0.19% | 0.34% | 0.35% | 0.15% |
|  | P | 0.74% | 0.85% | 0.94% | 0.34% | 0.34% | 0.16% | 0.25% | 0.85% | 0.46% | 0.24% | 0.52% | 0.75% | 0.64% | 0.19% | 0.45% |
